# Angiopoietin-Like 7 Is an Anti-Angiogenic Protein Required to Prevent Vascularization of the Cornea

**DOI:** 10.1371/journal.pone.0116838

**Published:** 2015-01-26

**Authors:** Tetsuya Toyono, Tomohiko Usui, Seiichi Yokoo, Yukako Taketani, Suguru Nakagawa, Masahiko Kuroda, Satoru Yamagami, Shiro Amano

**Affiliations:** 1 Department of Ophthalmology, Graduate School of Medicine, University of Tokyo, Tokyo, Japan; 2 Department of Pathology, Tokyo Medical University, Tokyo, Japan; University of Minnesota Medical School, UNITED STATES

## Abstract

**Purpose:**

We sought to identify the anti-angiogenic molecule expressed in corneal keratocytes that is responsible for maintaining the avascularity of the cornea.

**Methods:**

Human umbilical vein endothelial cells (HUVECs) were cultured with either human dermal fibroblasts or with human corneal keratocytes under serum-free conditions. The areas that exhibited blood vessel formation were estimated by immunostaining the cultures with an antitibody against CD31, a blood vessel marker. We also performed microarray gene-expression analysis and selected one molecule, angiopoietin-like 7 (ANGPTL7) for further functional studies conducted with the keratocytes and *in vivo* in mice.

**Results:**

Areas showing blood vessel formation in normal serum-free medium were conditions were markedly smaller when HUVECs were co-cultured with corneal keratocytes than when they were co-cultured with the dermal fibroblasts under the same conditions. Microarray analysis revealed that *ANGPTL7* expression was higher in keratocytes than in dermal fibroblasts. *In vitro*, inhibiting *ANGPTL7* expression by using a specific siRNA led to greater tube formation than did the transfection of cells with a control siRNA, and this increase in tube formation was abolished when recombinant *ANGPTL7* protein was added to the cultures. *In vivo*, intrastromal injections of an *ANGPTL7* PshRNA into the avascular corneal stroma of mice resulted in the growth of blood vessels.

**Conclusions:**

*ANGPTL7*, which is abundantly expressed in keratocytes, plays a major role in maintaining corneal avascularity and transparency.

## Introduction

The cornea is present on most of the surface of the eyeball, and this transparent tissue is avascular. Corneal avascularity is the result of an active process involving the production of anti-angiogenic factors that counterbalance pro-angiogenic and pro-inflammatory factors. For example, corneal epithelial cells express soluble vascular endothelial growth factor receptor-1 (sVEGFR1) to neutralize VEGF activity, even in normal corneas [1]. Keratocytes are the predominant cellular components of the corneal stroma and are similar to fibroblasts. When keratocytes are activated by pathological conditions such as trauma and inflammation, these cells often transform into myofibroblasts, which produce an extracellular matrix, collagen degrading enzymes, and cytokines [2]. These factors orchestrate the cellular behavior that is associated with the development of corneal neovascularization, which is one of the major causes of the loss of corneal transparency [2, 3]. Conversely, in the normal cornea, the keratocytes are quiescent [3] and likely maintain the corneal transparency and avascularity. However, the contributions of keratocytes to the preservation of corneal avascularity have not yet been fully elucidated.

Angiogenesis is a complex biological process involving cell-to-cell interactions between the vessel-forming endothelial cells and the host microenvironment. Previously, an i*n vitro* system for co-culturing human umbilical vein endothelial cells (HUVECs) and human dermal fibroblasts (HDFs) was previously established to mimic the anigiogenic microenvironment, and it was used as a model system to explore the growth factors and regulatory networks that control angiogenesis [4]. This system relies on fibroblasts secreting the necessary matrix components that act as a scaffold for tube formation [4]. In this study, we first attempted to establish the co-culture system by using HUVECs and cultivated human corneal keratocytes (HCKs) in order to examine the properties of keratocytes that contributes to blood vessel tube formation. Next, we performed microarray analysis to compare the gene-expression profiles of the HCKs with that of the HDFs to identify potential anti-angiogenic genes in the HCKs. Lastly, the function of one molecule identified from the microarray analysis, angiopoietin-like 7 (ANGPTL7), was examined both *in vitro* and *in vivo*.

## Materials and Methods

### Cell culture

Primary HDFs and HUVECs were purchased from Lonza (Allendale, NJ, USA). HDFs were seeded in culture dishes with Dulbecco’s modified Eagle medium (DMEM)/F12 containing 2% fetal bovine serum (FBS). The medium was changed every two days until the cells reached confluence. Primary HCKs were isolated from a corneoscleral button retrieved from an eye bank (SightLife, Seattle, WA, USA) as previously described [5, 6]. Briefly, the corneal epithelium was removed from the stroma by scraping with a razor blade, and the Descemet’s membrane and endothelial cells were peeled away separately as a sheet by using fine forceps. A central corneal button was cut using an 8.0 mm trephine (Biopsy Punch, Kai Medical, Gifu, Japan), and subsequently incubated overnight at 37°C in a basal medium DMEM/ F12 medium supplemented with B27 (Invitrogen, Carlsbad, CA, USA) containing 0.02% collagenase (Sigma-Aldrich, St. Louis, MO, USA). Next, the digested tissue and cells were dispersed by pipetting, and then centrifuged at 800 x *g* for 5 min. After removing the supernatant, the keratocytes were resuspended in 1.0 mL of the basal culture medium and seeded into culture dishes. The medium was changed every two days until the cells reached confluence. Second-passage cells were used in the co-culture assays.

### Co-culture tube formation assay

HDFs and HCKs were seeded in 24-well plates (4 × 10^4^ cells/well), and cultured in DMEM/F12 containing 2% FBS for five days. The medium was then changed to DMEM/F12 supplemented with serum free B27 supplementation for two days. On day seven, HUVECs were seeded onto confluent HDFs or HCKs (2 × 10^4^ cells/well), and these co-cultures were grown in the DMEM/F12 supplemented with serum-free B27 for an additional 11 days. The formation of blood vessel tubes by the HUVECs was quantified by immunohistochemistry with the blood vessel marker, CD31. On day seven, the number of cells in several wells was determined by staining them with 4′,6-diamidino-2-phenylindole dihydrochloride (DAPI) and performing a nonradioactive colorimetric assay with water-soluble Tetrazolimum salt (WST-1); Takara Bio, Inc., Shiga, Japan).

### *In vitro* immunohistochemistry *in vitro*

Tube formation was quantified by using a tubule-staining kit (Kurabo, Okayama, Japan) as per the manufacturer’s protocol. Briefly, on day 18, co-cultured cells were fixed in 70% ice-cold ethanol for 30 min at room temperature. Next, the cells were washed thrice in blocking solution (phosphate buffered saline (PBS) with 1% bovine serum albumin) and then incubated with a monoclonal anti-human CD31 antibody (1:4000 dilution; Kurabo) and incubation at 37°C for 60 min. The cells were again washed with blocking solution and then incubated with a secondary antibody (1:500, goat anti-mouse IgG conjugated to alkaline phosphatase; Kurabo) at 37°C for 60 min. The immunoreactivity was detected by incubating cells for 5 min with 5-bromo-4-chloro-3-indolyl-phosphate/nitro blue tetrazolium (BCIP/NBT, Kurabo).

Quantitative image analysis of the CD31 immunoreactivity was conducted using NIH ImageJ software (http://rsb.info.nih.gov/ij/). Twelve fields (40x magnification) of each group were chosen in a randomly for the analysis. All compared images were captured using the same exposure and all photography settings were used at under-saturation levels. The images of the immunostained cells (CD31-positive cells) were transformed to gray-scale images and an optical density plot of the selected areas was generated using the Histogram tool. The immunostaining area (arbitrary unit, AU) was calculated as the difference between membrane or cytoplasm immunostaining and the background. Lastly, the area was normalized using the pixel area. All of the data were analyzed using the statistical software JMP Pro version 10.0.2 (SAS Institute Inc., Cary, NC, USA), and the Mann-Whitney U test was used for comparing the differences.

### WST-1 assay

To confirm the cellular viabilities of the HDFs and HCKs, a WST-1 assay (Takara Bio) was performed as described previously. Briefly, the WST-1 reagent was applied to the confluent HDFs and HCKs in DMEM/F12 medium, and after incubation at 37°C for 1 h, the dye intensity at 450 nm was measured using a plate reader (Victor 3V Multilabel Counter Model 1420; Perkin Elmer, Waltham, MA, USA). The results were expressed as the mean percentage of viable cells ± standard deviation (SD).

### Microarray analysis

HDFs and HCKs were seeded at 4 × 10^4^ cells/well in 24-well tissue culture plates and cultured in DMEM/F12 supplemented with serum-free B27 for 48h. Total RNA was extracted from the cells by using Isogen (Nippon gene, Toyama, Japan) as per the manufacturer’s instructions. Quantification and quality assessment of the isolated RNA samples were performed using a NanoDrop2000 (Thermo Scientific, Waltham, MA, USA) in accordance with the manufacturer’s instructions. A260/A230 and A260/A280 ratios between 1.8 and 2.1 were confirmed in all RNA samples. RNA was amplified into cRNA and labeled according to the Agilent One-Color Microarray Based Gene Expression Analysis protocol (Agilent Technologies, Palo Alto, CA, USA). Global gene-expression analysis was performed using a SurePrint G3 human gene expression 8×60 microarray kit (G4851A, Agilent Technologies) which contained 27,958 Entrez Gene RNAs and 7419 large intergenic noncoding RNAs (lincRNAs). To confirm the results of microarray analysis, we performed quantitative RT-PCR (qRT-PCR) and western blot analysis.

### qRT-PCR

Total RNA was extracted from the cells or corneas by using Isogen, and cDNA was synthesized using reverse transcriptase (PrimerScript RT Master Mix, Takara Bio). qRT-PCR was performed using SYBR green master mix reagents (SYBR Premix Ex Taq (Tli RNase H Plus), Takara Bio) with the following temperature profile; 95°C for 5 sec, followed by 40 cycles of 95°C for 5 sec and 60°C for 30 sec. Total reaction volume was 25 mL including SYBR green master mix (Takara Bio) with cDNA transcribed from 100 ng of RNA and 0.4 mM forward and reverse primers (Table 1). The comparative Ct (ΔΔCT) method was used to compare the mRNA expression levels of the genes of interest. The gene encoding glyceraldehyde-3-phosphate dehydrogenase (*GAPDH*) was chosen as an internal control.

**Table 1:** Primer sets for qPCR analysis.

**Target gene**	**Sequence**
Human ANGPTL1	forward, 5′- GGATTCTATGATGTGGCATAATGGT −3′
	reverse, 5′- AATTCCATCTTGGTGCTTGCT −3′
Human ANGPTL2	forward, 5′- GGAGGTTGGACTGTCATCCAGAG −3′
	reverse, 5′- GCCTTGGTTCGTCAGCCAGTA −3′
Human ANGPTL3	forward, 5′- ACAGTTCCACGTTGCTTGAA −3′
	reverse, 5′- CCCAACTGAAGGAGGCCATT −3′
Human ANGPTL4	forward, 5′- GGACAAGAACTGCGCCAAGAG −3′
	reverse, 5′- AGGTTGGAATGGCTGCAGGT −3′
Human ANGPTL5	forward, 5′- TTAAGGATACCATTGGCTCTGTCAC −3′
	reverse, 5′- CCTCTGGAAATCAATTATCCCATCA −3′
Human ANGPTL6	forward, 5′- CAGTACCATGGTGATGCTGGAGA −3′
	reverse, 5′- AGTGGGCACAGGCATGGTA −3′
Human ANGPTL7	forward, 5′- CGGCTGCGTGTAGAGATGGA −3′
	reverse, 5′- CCTTGGTGCTGAAGGCTGTGT −3′
Human AKR1B10	forward, 5′- GGCAACCATACTCAGCTTCAACAG −3′
	reverse, 5′- GGGACATGAGTGGAGGTAGTCACA −3′
Human GRP	forward, 5′- TTTGCTGGGTCTCATAGAAGCAAAG −3′
	reverse, 5′- TCACGTTGAGAACCTGGAGCA −3′
Human PIP	forward, 5′- GCTTGCTCCAGCTCCTGTT −3′
	reverse, 5′- TGGACGTACTGACTTGGGAATG −3′
Human COL1A1	forward, 5′- GCTTGCTCCAGCTCCTGTT −3′
	reverse, 5′- TGGACGTACTGACTTGGGAATG −3′
Human GAPDH	forward, 5′- TTGATTTTGGAGGGATCTCG −3′
	reverse, 5′- GAGTCAACGGATTTGGTCGT −3′
Mouse ANGPTL7	forward, 5′- AATGAACATATCCACCGGCTCAC −3′
	reverse, 5′- AGTTCATTGCCCAACGCAAAG −3′
Mouse GAPDH	forward, 5′-CACATTGGGGGTAGGAACAC−3′
	reverse, 5′-AACTTTGGCATTGTGGAAGG−3′

### Western blot analysis

Samples of the culture media were also collected to determine the ANGPTL7 production. An equal volume of the SDS sample buffer (Lane Marker Reducing Sample Buffer; Thermo Scientific, Waltham, MA, USA) was added to the collected medium, mixed and boiled for 5min. The samples (50 mg/lane) were electrophoresed on 12% acrylamide gel (Bio-Rad, Hercules, CA, USA) and subsequently transferred to poly-vinylidene difluoride membranes (Bio Rad) for western blot analysis. Membranes were incubated with an anti-angptl7 polyclonal antibody (1:100; Abcam, Cambridge, UK) for 60 min at room temperature and immunostaining was detected using the alkaline phosphatase technique performed using a BCIP/NBT immunoblotting kit (Bio Rad).

### Suppression of Angptl7 expression *in vitro*

HCKs were seeded in 24-well tissue culture plates and seeded at 4 × 10^4^ cells/well in DMEM/F12 supplemented with 2%FBS and grown for 3 days. The *ANGPTL7* short interfering RNA (siRNA) (50 nM, sc-88201, Santa Cruz Biotechnology, Dallas, TX, USA) and Control siRNA (50 nM, sc-37007, Santa Cruz Biotechnology) were transfected into the cells by using Lipofectamine RNAiMAX (Invitrogen), and the cells were incubated for an additional 48 h. The medium was then replaced with DMEM/F12 supplemented with serum-free B27 for another 48 or 96 hours. Semiquantitative RT-PCR was performed using specific primers to monitor the *ANGPTL7* gene-expression knockdown.

We also transfected the *ANGPTL7* siRNA and Control siRNA into co-cultures of HCKs and HUVECs and then examined tube formation. The HUVECs were seeded onto confluent HCKs (20,000 cells/well) and were cultured in serum-free medium with *ANGPTL7* siRNA and Control siRNA (serum free) for 6 days. To several of the wells, we also added human recombinant *ANGPTL7* (500 ng/mL, 914-AN-025/CF, R&D systems, MN, USA). Tube formation by the HUVECs was then quantified by means of CD31 immunohistochemistry. Furthermore, we also measured the mRNA expression levels of other *ANGPTL* family members (1–6) after transfecting cells with *ANGPTL7* siRNA for 48 h.

### *In vivo* suppression of Angptl7 expression in mouse cornea

**Figure 1:**
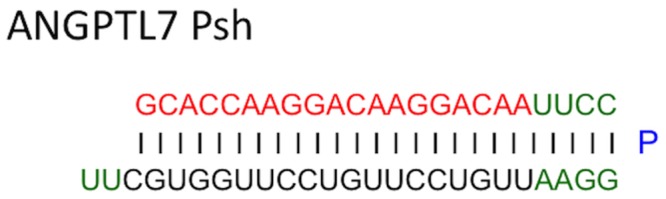
PshRNA platform. PshRNA was synthesized in solid phase as single-stranded RNAs that, following synthesis, self-anneal into a unique helical structure containing a single stem loop.

Under direct microscopic observation, a custom made 33-gauge needle (30° bevel) attached to a 5-mL syringe (ITO, Shizuoka, Japan) was manually introduced into the corneal stroma and advanced to the center, where 5 ml of 1 mM *ANGPTL7* PshRNA or Control PshRNA (Bonac, Fukuoka, Japan) was forcibly injected into the stroma. PshRNAs were synthesized in solid phase as single-stranded RNAs that, following synthesis, self-anneal into a unique helical structure containing a single stem loop (Fig. 1). This stem loop contained proline derivatives similar to PnkRNA as previously described [7]. Seven days after the injection, the corneas were harvested to quantify the corneal neovascularization.

### Quantification of hemangiogenesis *in vivo*

Hemangiogenesis was quantified by performing corneal whole-mount CD31 staining as previously described [8]. Briefly, following neovascularization, we obtained a corneal button that was excised 0.5mm posterior from the limbal vessel and rinsed in PBS, and then fixed in acetone for 10 min at-20°C. Subsequently, the corneas were washed thrice in PBS, blocked with 3% BSA in PBS for 48 h at room temperature, stained with a rat anti-mouse CD31 antibody (BD Biosciences, Franklin Lakes, NJ) and incubated at 4°C overnight. The corneas were again washed with PBS and stained with a secondary antibody (Alexa Fluor 594 donkey anti-rat IgG; Invitrogen) for 3 h at room temperature. The stained whole mounts of corneas were placed on glass slides and examined under a fluorescence microscope (BZ-9000; Keyence, Osaka, Japan). ImageJ was used for image analysis. Neovascularization was quantified by setting a threshold level of CD31-positive fluorescence, above which only vessels were depicted. Neovascularization was quantified in a masked manner. The vascularization area was outlined by using the innermost vessel of the limbal arcade as the border, and the surface area of corneal neovascularization (vascularized area/total corneal area) was quantified.

### Ethics statement

This study adhered to the Association for Research in Vision and Ophthalmology Statement for the Use of Animals in Ophthalmic and Vision Research and was approved by the University of Tokyo Hospital Animal Care Committee. The animals were allowed free access to food and water. A 12-h light-dark cycle was maintained. All surgical procedures were performed under general anesthesia by injecting the mice (8-week-old males C57BL/6) with xylazine hydrochloride (5mg/kg of body weight) and ketamine hydrochloride (35mg/kg of body weight). The animals were euthanized by an intraperitoneally injecting them with Nembutal (60 mg/kg of body weight) (Sigma) before harvesting the eyes. All efforts were made to minimize suffering.

### Statistical analysis

All of the data were analyzed using the statistical software (JMP Pro version 10.0.2), and values of *p* < 0.05 were considered statistically significant.

## Results

### Tube formation was weaker on corneal cells than on dermal fibroblasts

The HCKs and HDFs were cultured for 7 days and then HUVECs were co-cultured with them. Before the adding HUVECs to HCKs and HDFs, the numbers of the HCKs and HDFs at confluence were not significantly different, as shown by DAPI staining (cell counts/field; HCKs 158.2±15.2, HDFs 149.8±18.7, *p* = 0.12). Moreover, results of the WST-1 assay revealed that the viability the HCKs and HDFs at confluence did not differ significantly (relative viabilities, HCKs:HDFs = 1:0.94; *p* = 0.43).

**Figure 2:**
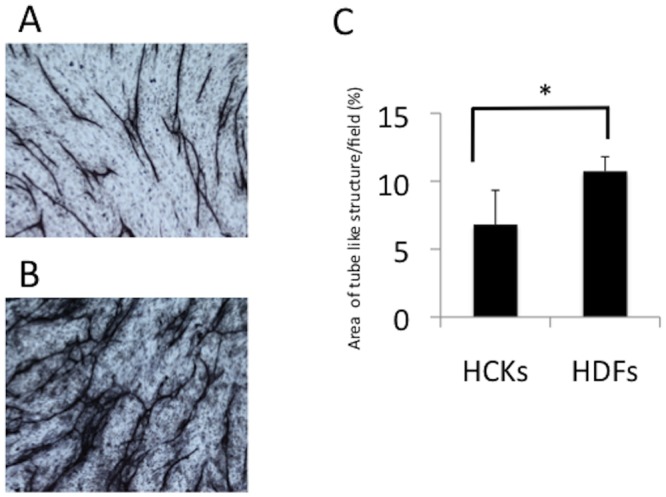
Tube formation by HUVECs co-cultured with corneal cells or dermal fibroblasts under serum-free conditions. The areas of tube formation by HUVECs under the normal serum-free medium conditions were 6.81 ± 1.07% in co-culture with the human corneal keratocytes (HCKs, A) and 10.37 ± 2.52% in co-culture with the human dermal fibroblasts (HDFs, p < 0.0001, B and C). N = 12 per condition.

We assessed tube formation in both co-culture models by immunostaining the cultures with an anti-CD31 monoclonal antibody. The areas of tube formation by HUVECs under the normal serum-free medium conditions were 6.81 ± 1.07% in co-culture with HCKs and 10.37 ± 2.52% in the co-culture with HDFs (p < 0.0001, Fig. 2). This result suggested that HCKs provided a more anti-angiogenic environment for the HUVECs than did the HDFs.

### Microarray gene expression analysis

**Figure 3:**
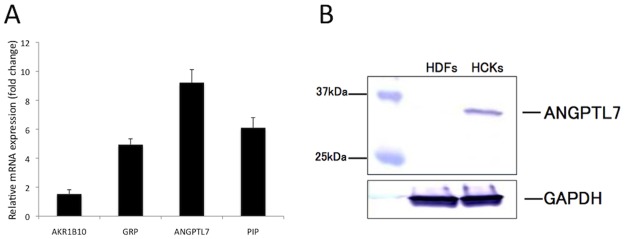
Relative expression of ANGPTL7 in HCKs and HDFs. *AKR1B10, GRP, ANGPTL7*, and *PIP* levels were elevated in the cDNA derived from HCKs as compared to with that from HDFs (A). In western blot analysis, ANGPTL7 was detected in culture media collected from HCKs but not i HDFs (B). N = 3 per condition.

To investigate the difference between the tube-formation responses in the two co-culture models, we performed microarray analysis. Table 2 shows the genes that were most upregulated in HCKs relative to those in HDFs; the Supplemental Data section lists 100 upregulated and downregulated genes in HCKs. We performed qRT-PCR to confirm the differential expression of several genes identified by means of microarray analysis. The expression of *AKR1B10, GRP, ANGPTL7*, and *PIP* was higher in HCKs than in HDFs (Fig. 3A). Furthermore, in western blot analysis, *ANGPTL7* was detected in the culture medium collected from the HCKs but not the HDFs (Fig. 3B).

**Table 2:** Upregulated genes in HCKs relative to those in the HDFs.

**Official Full Name**	**Gene Symbol**	**Fold change**	**Location**	**Unigene ID**
aldo-keto reductase family 1, member B10	AKR1B10	13.38	hs|7q33	Hs.116724
gastrin-releasing peptide	GRP	12.78	hs|18q21.32	Hs.153444
angiopoietin-like 7	ANGPTL7	12.34	hs|1p36.22	Hs.146559
aldo-keto reductase family 1, member B15	AKR1B15	12.22	hs|7q33	Hs.116724
prolactin-induced protein	PIP	10.06	hs|7q34	Hs.99949
cancer/testis antigen 1A (CTAG1A)	CTAG1A	9.77	hs|Xq28	Hs.534310
carbohydrate (N-acetylglucosamine 6-O) sulfotransferase 6	CHST6	9.46	hs|16q23.1	Hs.655622
transmembrane 4 L six family member 1 (TM4SF1)	TM4SF1	9.40	hs|3q25.1	Hs.351316
secretogranin II	SCG2	9.36	hs|2q36.1	Hs.516726
cytokine-like 1	CYTL1	9.34	hs|4p16.2	Hs.13872
ATPase, aminophospholipid transporter (APLT), class I, type 8A, member 1	ATP8A1	9.08	hs|4p13	Hs.435052
solute carrier family 4, sodium bicarbonate cotransporter, member 4	SLC4A4	9.01	hs|4q13.3	Hs.5462
transmembrane protein 155 (TMEM155)	TMEM155	8.53	hs|4q27	Hs.27524
Glutathione S-transferase theta 1	GSTT1	8.30	hs|22q11.23	Hs.268573
sushi domain containing 2	SUSD2	8.01	hs|22q11.23	Hs.131819
aldehyde dehydrogenase 3 family, member A1	ALDH3A1	7.78	hs|17p11.2	Hs.531682
nuclear receptor subfamily 0, group B, member 1	NR0B1	7.78	hs|Xp21.2	Hs.268490
transcription factor AP-2 beta (activating enhancer binding protein 2 beta)	TFAP2B	7.67	hs|6p12.3	Hs.33102
hydroxysteroid (11-beta) dehydrogenate 1	HSD11B1	7.51	hs|1q32.2	Hs.195040
sclerostin	SOST	7.07	hs|17q21.31	Hs.349204

### Inhibition of ANGPTL7 increased tube formation in the HCK-HUVEC co-culture model

**Figure 4:**
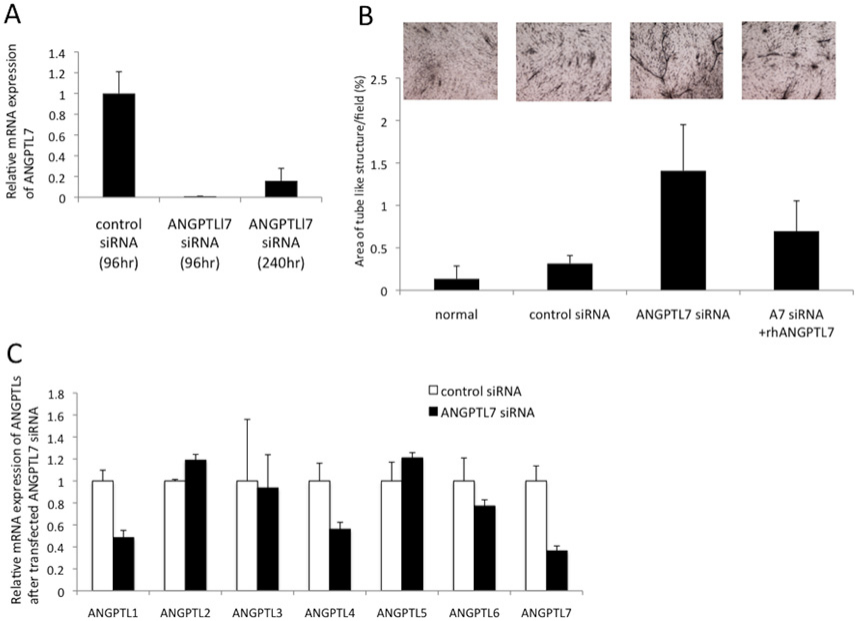
*ANGPTL7* siRNA tranfection increased tube formation in HCK-HUVEC co-culture. *ANGPTL7* mRNA expression was drastically inhibited following the administration of *ANGPTL7* siRNA for up to 240 h after transfection (A). The tube-formation areas under the normal serum-free medium (normal) and control siRNA treatment were 0.13 ± 0.15% and 0.31 ± 0.09%, respectively (B). In the cells transfected with the *ANGPTL7* siRNA the tube formation areas increased significantly (1.41 ± 0.54%, *p* < 0.001, B). This siRNA effect was potently suppressed when human recombinant ANGPTL7 (A7 siRNA+rhANGPTL7) was added to the cells (0.69 ± 0.30%, *p* < 0.001, B). N = 12 per condition. In the cells in which *ANGPTL7* expression was suppressed, the mRNA expression levels of *ANGPTL1* and *ANGPTL4* were also decreased (by 48.4% and 56.1%, respectively), but the expression of *ANGPTL2* was increased (119%). N = 2 per each condition.

To elucidate the function of *ANGPTL7* in angiogenesis, we analyzed the tube formation by the HUVECs co-cultured with HCKs that had been transfected with an *ANGPTL7* siRNA. *ANGPTL7* mRNA expression was drastically inhibited following the administration of the *ANGPTL7* siRNA, even at 240 h after transfection (Fig. 4A). The areas of tube formation in normal serum-free medium and after control siRNA treatment were 0.13 ± 0.15% and 0.31 ± 0.09%, respectively (Fig. 4B), but when the cells were transfected with the *ANGPTL7* siRNA, the areas of tube formation were significantly increased when compared with the areas in the control siRNA group (1.41 ± 0.54%, p < 0.001, Fig. 4B). However, this effect of the *ANGPTL7* siRNA was significantly suppressed when human recombinant ANGPTL7 was added to the cells (0.69 ± 0.30%, *p* < 0.001, compared with the ANGPTL7 siRNA group; Fig. 4B).

We also analyzed whether the mRNA expression of other *ANGPTL* family members was altered after the suppression of *ANGPTL7* expression. The expression levels of *ANGPTL1* and *ANGPTL4* were decreased to < 60% relative to the control level, whereas the expression of *ANGPTL2* was increased by nearly 120% compared with control (Fig. 4C).

### Blood vessel growth on the avascular corneas of mice after suppression of gene expression by using an ANGPTL7 PshRNA

**Figure 5:**
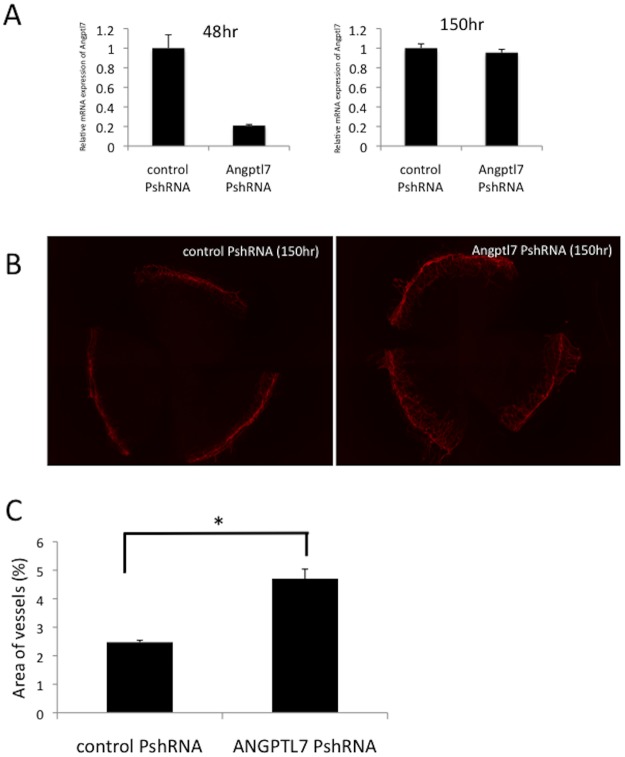
Blood vessel growth on the avascular corneas of mice after suppression of gene expression by using an *ANGPTL7* PshRNA. *ANGPTL7* mRNA expression was inhibited by almost 80% inhibition at 48 h after intrastomal injection of the *ANGPTL7* PshRNA, but normal expression was restored by 150 h (A, n = 3 per condition). The corneas of mice injected intrastromally with the control PshRNA did not exhibit any expression change (B, left panel). Notably, intrastromal injection of the *ANGPTL7* PshRNA resulted in the spontaneous development of blood vessel growth from the limbal areas in the avascular mouse corneas by day 4 after the injection (B, right panel). Areas of vessels in the mouse corneas were significantly different following the injection of control PshRNA injection and *ANGPTL7* PshRNA injection (*p* < 0.001, C, n = 4 per condition)

Next, to confirm the results derived from the tube formation assay *in vitro*, the hemangiogenic responses in mice treated with *ANGPTL7* PshRNA were investigated. The *ANGPTL7* mRNA expression was dramatically inhibited (almost 80% inhibition) by the intrastomal injection of the *ANGPTL7* PshRNA on 48 h after injection, but had been restored by 150 h (Fig. 5A). By contrast, the corneas of mice injected with the control PshRNA did not exhibit any alteration (Fig. 5). Notably, the intrastromal injection of the *ANGPTL7* PshRNA resulted in the spontaneous development of blood vessel growth from the limbal areas in the avascular mouse corneas by day 4 after the injection (Fig. 5).

## Discussion

In this study, we demonstrated that blood vessel formation by HUVECs in co-cultured together with corneal cells under serum-free conditions was considerably weaker than that by HUVECs co-cultured with dermal fibroblasts. These results suggested that HCKs potentially provide an anti-angiogenic environment for the HUVECs. Microarray analysis revealed that *ANGPTL7* was abundantly expressed in HCKs in comparison to HDFs. The suppression of *ANGPTL7* expression resulted in an angiogenic response both *in vitro* and *in vivo*. These results suggested that *ANGPTL7*, which is abundantly expressed in corneal cells, might be responsible for maintaining the corneal avascularity.

The ANGPTLs comprise a family of proteins structurally related to the angiopoietins [9]. ANGPTLs contain an N-terminal coiled-coil domain and a C-terminal fibrinogen-like domain, but, ANGPTLs do not bind to the angiopoietin receptor Tie1 or Tie2 [9]. *ANGPTL7* was originally named corneal-derived transcript 6 (CDT6) [10], and it is still considered an orphan ligand. Because stable transfection of human melanoma cells with *ANGPTL7*/*CDT6* led to a reduction of tumor growth in a mouse xenograft model relative to the growth observed in controls, ANGPTL7 was speculated to function as a negative regulator of angiogenesis and to possibly contribute to corneal avascularity [10]. However, subsequent studies by Bouis et al demonstrated that *ANGPTL7*/*CDT6* had no effect on tumor growth [11, 12], casting doubt on the angiogenic properties of this gene. Our results have shown that siRNA-mediated suppression of *ANGPTL7* expression led to blood vessel formation on HCKs and this was coupled with a change in the expression of other ANGPTLs (Fig. 4); When we suppressed *ANGPTL7* expression *in vitro* using the siRNA, the expressions of *ANGPTL1* and *ANGPTL 4* were also decreased but that of *ANGPTL2* was increased (Fig. 4C). ANGPTL1 and ANGPTL4 have been reported to function as anti-angiogenic factors under certain conditions [13]. Guo et al suggested that ANGPTL4 exerts anti-angiogenic effects under physiological conditions [14]. By contrast, ANGPTL2 has been considered to function as a pro-angiogenicfactor [8]. Therefore, this findings might partially explain why ANGPTL7 suppression could accelerate tube formation *in vitro* (Fig. 4). Furthermore, although tube formation was decreased following the addition of recombinant ANGPTL7 (Fig. 4), this reversal effect of ANGPTL7 was not observed in the HDF-HUVEC co-culture model (data not shown). The expression of *ANGPTL7* was observed to be elevated in the corneal tissue, but not in other tissues such as brain, liver, and heart [15], further suggesting that the corneal environment might be critical for normal ANGPTL activity. This variation in ANGPTL7 activity could result from the the lack of expression of its receptor in tissues other than the corneal tissue.

The expression of *ANGPTL7* has also been observed in the trabecular meshwork, which is located adjacent to the cornea and is the drainage site for aqueous humor [16, 17]. This tissue is also avascular and the vessel invasion to the trabecular meshwork in hypoxic retinal diseases such as diabetic retinopathy is observed in the form of severe glaucoma (neovascular glaucoma). Therefore, the expression of *ANGPTL7* in the trabecular meshwork might also play a crucial role in maintaining its avascularity. Furthermore, *ANGPTL7* expression was upregulated together with that of type I collagen following the addition of dexamethasone to cultured human trabecular meshwork cells [16–20]. Type I collagen is widely recognized one of the main components of the corneal stroma and the application of topical corticosteroids inhibits corneal neovascularization [21, 22]. The mechanisms by which ANGPTL7 exerts anti-angiogenic effects have thus far remained unknown. Because type I collagen retards tube formation *in vitro* [23], the assembly of dense type I collagen fibers in the corneal stroma might prevent the entry of vessel sprouts into avascular corneas. Thus, we speculate that the synthesis of type I collagen stimulated by *ANGPTL7* might serve as one possible mechanism by which corneal avascularity is maintained. In light of our results, it is possible that application of a topical corticosteroid inhibits corneal neovascularization through the upregulation of *ANGPTL7* and type I collagen expression.

In this study, we intrastromally injected a PshRNA in order to suppress the expression of the *ANGPTL7* in mice. Although siRNAs are widely used in typical RNA interference experiments, siRNAs are double stranded and might act as ligands for toll-like receptor 3, which could induce inflammatory reactions. The PshRNA used in our *in vivo* studies, is a single-stranded RNA. This short hairpin single-stranded RNA does not induce IFN-gamma inflammatory reactions (data not shown). Thus, our treatment procedure might be capable of inducing corneal neovascularization without stimulating an inflammatory response that is observed when other reagents are used. Furthermore, this drug delivery system might facilitate the development of a novel *in vivo* RNA interference-based therapeutic strategy.

A key feature of our *in vitro* study is that we could use it to observe tube formation under non-serum conditions. HUVECs typically cannot grow by themselves under serum-free conditions. In the original co-culture system developed using fibroblasts and HUVECs, allogenic serum such as FBS was used [4]. Because FBS contains various growth factors and hormones, variations among FBS lots could present a problem. Another potential limitation of the system is that observing the pure effects of the various factors on tube formation might be challenging. Thus, a non-serum co-culture system would be an ideal tool for investigating gene-expression profiles and screening for drugs in the case of angiogenesis, and this could lead to the development of an angiogenesis assay.

The results of our co-culture assays suggested that HCKs express fewer pro-angiogenic factors and/or more anti-angiogenic factors than do HDFs. Although the anti-angiogenic properties of keratocytes remain to be fully elucidated, keratocytes have been reported to potentially express anti-angiogenic molecules [24, 25]. For example, although keratocytes expressed thrombospondin, which is widely recognized as an anti-angiogenic molecule, the expression of this molecule was attenuated following the infection of cultured keratocytes with herpes simplex virus-1 [24]. Based on using *in vitro* assays, Azar et al also reported that keratocyte-derived membrane-type matrix metalloproteinase exerts potent anti-angiogenic effects [25]. Taken together with our results, this suggests that keratocytes likely protect against corneal neovascularization.

In conclusion, we determined that *ANGPTL7*, which is abundantly expressed in keratocytes, plays a major role in maintaining corneal avascularity, and that the corneal environment might be crucial for its activity. Because the receptor of *ANGPTL7* has not yet been identified, in future studies, it will be critical to explore ANGPTL7 signaling pathways and the molecular mechanisms of its action in corneal cells.

## Supporting Information

S1 Table Relative up-regulated genes in HCKs. (PDF)

S2 Table Relative down-regulated genes in HCKs. (PDF)
