# Supplementary material for: Angiopoietin-Like 7 Is an Anti-Angiogenic Protein Required to Prevent Vascularization of the Cornea
Source: PLoS One. 2015 Jan 26;10(1):e0116838. doi: 10.1371/journal.pone.0116838 (PMC4306551; doi:10.1371/journal.pone.0116838)
Supplement: S1 Table — (PDF) [file pone.0116838.s001.PDF]

Relative upregulated genes in HCKs

Official Full Name	Gene Symbol	Fold change	Location	Unigene ID
Homo sapiens aldo-keto reductase family 1, member B10 (aldose reductase) (AKR1B10), mRNA [NM_020299]	AKR1B10	13.38	hs|7q33	Hs.116724
Homo sapiens gastrin-releasing peptide (GRP), transcript variant 1, mRNA [NM_002091]	GRP	12.78	hs|18q21.32	Hs.153444
Homo sapiens angiopoietin-like 7 (ANGPTL7), mRNA [NM_021146]	ANGPTL7	12.34	hs|1p36.22	Hs.146559
Homo sapiens aldo-keto reductase family 1, member B15 (AKR1B15), mRNA [NM_001080538]	AKR1B15	12.22	hs|7q33	Hs.116724
Homo sapiens prolactin-induced protein (PIP), mRNA [NM_002652]	PIP	10.06	hs|7q34	Hs.99949
Homo sapiens cancer/testis antigen 1A (CTAG1A), mRNA [NM_139250]	CTAG1A	9.77	hs|Xq28	Hs.534310
Homo sapiens carbohydrate (N-acetylglucosamine 6-O) sulfotransferase 6 (CHST6), mRNA [NM_021615]	CHST6	9.46	hs|16q23.1	Hs.655622
Homo sapiens transmembrane 4 L six family member 1 (TM4SF1), mRNA [NM_014220]	TM4SF1	9.40	hs|3q25.1	Hs.351316
Homo sapiens secretogranin II (SCG2), mRNA [NM_003469]	SCG2	9.36	hs|2q36.1	Hs.516726
Homo sapiens cytokine-like 1 (CYTL1), mRNA [NM_018659]	CYTL1	9.34	hs|4p16.2	Hs.13872
Homo sapiens ATPase, aminophospholipid transporter (APLT), class I, type 8A, member 1 (ATP8A1), transcript variant 1, mRNA [NM_006095]	ATP8A1	9.08	hs|4p13	Hs.435052
Homo sapiens solute carrier family 4, sodium bicarbonate cotransporter, member 4 (SLC4A4), transcript variant 2, mRNA [NM_003759]	SLC4A4	9.01	hs|4q13.3	Hs.5462
Homo sapiens transmembrane protein 155 (TMEM155), mRNA [NM_152399]	TMEM155	8.53	hs|4q27	Hs.27524
Homo sapiens glutathione S-transferase theta 1 (GSTT1), mRNA [NM_000853]	GSTT1	8.30	hs|22q11.23	Hs.268573
Homo sapiens sushi domain containing 2 (SUSD2), mRNA [NM_019601]	SUSD2	8.01	hs|22q11.23	Hs.131819
Homo sapiens aldehyde dehydrogenase 3 family, member A1 (ALDH3A1), transcript variant 2, mRNA [NM_000691]	ALDH3A1	7.78	hs|17p11.2	Hs.531682
Homo sapiens nuclear receptor subfamily 0, group B, member 1 (NR0B1), mRNA [NM_000475]	NR0B1	7.78	hs|Xp21.2	Hs.268490
Homo sapiens transcription factor AP-2 beta (activating enhancer binding protein 2 beta) (TFAP2B), mRNA [NM_003221]	TFAP2B	7.67	hs|6p12.3	Hs.33102
Homo sapiens hydroxysteroid (11-beta) dehydrogenase 1 (HSD11B1), transcript variant 2, mRNA [NM_181755]	HSD11B1	7.51	hs|1q32.2	Hs.195040
Homo sapiens sclerostin (SOST), mRNA [NM_025237]	SOST	7.07	hs|17q21.31	Hs.349204
Homo sapiens prostaglandin-endoperoxide synthase 2 (prostaglandin G/H synthase and cyclooxygenase) (PTGS2), mRNA [NM_000963]	PTGS2	6.86	hs|1q31.1	Hs.196384
Homo sapiens phospholipase A2, group IVA (cytosolic, calcium-dependent) (PLA2G4A), mRNA [NM_024420]	PLA2G4A	6.65	hs|1q31.1	Hs.497200
Homo sapiens chitinase 3-like 2 (CHI3L2), transcript variant 3, mRNA [NM_001025199]	CHI3L2	6.55	hs|1p13.3	Hs.514840
Homo sapiens serpin peptidase inhibitor, clade A (alpha-1 antiproteinase, antitrypsin), member 5 (SERPINA5), mRNA [NM_000624]	SERPINA5	6.52	hs|14q32.13	Hs.159628
Homo sapiens phosphatidic acid phosphatase type 2 domain containing 1A (PPAPDC1A), mRNA [NM_001030059]	PPAPDC1A	6.41	hs|10q26.12	Hs.40479
Homo sapiens thiosulfate sulfurtransferase (rhodanese)-like domain containing 1 (TSTD1), transcript variant 1, mRNA [NM_001113207]	TSTD1	6.27	hs|1q23.3	Hs.720030
Homo sapiens chondroitin sulfate synthase 3 (CHSY3), mRNA [NM_175856]	CHSY3	6.25	hs|5q23.3	Hs.213137
Homo sapiens carbohydrate (keratan sulfate Gal-6) sulfotransferase 1 (CHST1), mRNA [NM_003654]	CHST1	6.12	hs|11p11.2	Hs.104576
Homo sapiens elongation of very long chain fatty acids (FEN1/Elo2, SUR4/Elo3, yeast)-like 2 (ELOVL2), mRNA [NM_017770]	ELOVL2	6.11	hs|6p24.2	Hs.656436
Homo sapiens ABI family, member 3 (NESH) binding protein (ABI3BP), mRNA [NM_015429]	ABI3BP	6.07	hs|3q12.2	Hs.477015
Homo sapiens nebulette (NEBL), transcript variant 1, mRNA [NM_006393]	NEBL	6.02	hs|10p12.31	Hs.5025
Homo sapiens prolyl 4-hydroxylase, alpha polypeptide III (P4HA3), mRNA [NM_182904]	P4HA3	5.99	hs|11q13.4	Hs.660541
Homo sapiens thrombospondin 4 (THBS4), mRNA [NM_003248]	THBS4	5.97	hs|5q14.1	Hs.211426
Homo sapiens F11 receptor (F11R), mRNA [NM_016946]	F11R	5.92	hs|1q23.3	Hs.517293
Homo sapiens hypothetical LOC100192378 (LOC100192378), non-coding RNA [NR_024360]	LOC100192378	5.91	hs|8q21.11	Hs.596420
Homo sapiens phosphodiesterase 4D, cAMP-specific (PDE4D), transcript variant 4, mRNA [NM_001197218]	PDE4D	5.91	hs|5q12.1	Hs.117545
Homo sapiens DIRAS family, GTP-binding RAS-like 3 (DIRAS3), mRNA [NM_004675]	DIRAS3	5.78	hs|1p31.3	Hs.194695
Homo sapiens serine/threonine/tyrosine kinase 1 (STYK1), mRNA [NM_018423]	STYK1	5.77	hs|12p13.2	Hs.24979
Homo sapiens UDP-N-acetyl-alpha-D-galactosamine:polypeptide N-acetylgalactosaminyltransferase 3 (GalNAc-T3) (GALNT3), mRNA [NM_004482]	GALNT3	5.65	hs|2q24.3	Hs.170986
Homo sapiens serum amyloid A2 (SAA2), transcript variant 1, mRNA [NM_030754]	SAA2	5.64	hs|11p15.1	Hs.1955
Homo sapiens cancer/testis antigen 2 (CTAG2), transcript variant 2, mRNA [NM_020994]	CTAG2	5.63	hs|Xq28	Hs.87225
Homo sapiens inositol polyphosphate-5-phosphatase, 145kDa (INPP5D), transcript variant 1, mRNA [NM_001017915]	INPP5D	5.62	hs|2q37.1	Hs.601911
Homo sapiens gastrin-releasing peptide receptor (GRPR), mRNA [NM_005314]	GRPR	5.51	hs|Xp22.2	Hs.567282
Homo sapiens delta/notch-like EGF repeat containing (DNER), mRNA [NM_139072]	DNER	5.45	hs|2q36.3	Hs.234074
Homo sapiens hypothetical LOC100192379 (LOC100192379), non-coding RNA [NR_024365]	LOC100192379	5.45	hs|4q27	Hs.607691
Homo sapiens multiple C2 domains, transmembrane 2 (MCTP2), transcript variant 1, mRNA [NM_018349]	MCTP2	5.44	hs|15q26.2	Hs.33368
Homo sapiens solute carrier family 7 (cationic amino acid transporter, y+ system), member 2 (SLC7A2), transcript variant 2, mRNA [NM_001008539]	SLC7A2	5.41	hs|8p22	Hs.448520
Homo sapiens serum amyloid A4, constitutive (SAA4), mRNA [NM_006512]	SAA4	5.39	hs|11p15.1	Hs.654493
Homo sapiens nuclear receptor subfamily 3, group C, member 2 (NR3C2), transcript variant 1, mRNA [NM_000901]	NR3C2	5.28	hs|4q31.23	Hs.163924
Homo sapiens chromosome 6 open reading frame 176 (C6orf176), transcript variant 1, non-coding RNA [NR_026860]	C6orf176	5.24	hs|6q27	Hs.31917
Homo sapiens Rh blood group, CcEe antigens (RHCE), transcript variant 1, mRNA [NM_020485]	RHCE	5.20	hs|1p36.11	Hs.523054
Homo sapiens hepatocyte growth factor (hepapoietin A; scatter factor) (HGF), transcript variant 2, mRNA [NM_001010931]	HGF	5.15	hs|7q21.11	Hs.396530
Homo sapiens membrane protein, palmitoylated 4 (MAGUK p55 subfamily member 4) (MPP4), mRNA [NM_033066]	MPP4	5.10	hs|2q33.1	Hs.63085
Homo sapiens cell adhesion molecule 3 (CADM3), transcript variant 1, mRNA [NM_021189]	CADM3	5.09	hs|1q23.2	Hs.365689
Homo sapiens chromosome 4 open reading frame 31 (C4orf31), mRNA [NM_024574]	C4orf31	4.99	hs|4q27	Hs.709520
Homo sapiens collectin sub-family member 11 (COLEC11), transcript variant 2, mRNA [NM_199235]	COLEC11	4.99	hs|2p25.3	Hs.32603
Homo sapiens vesicle amine transport protein 1 homolog (T. californica)-like (VAT1L), mRNA [NM_020927]	VAT1L	4.95	hs|16q23.1	Hs.461405
Homo sapiens retinoic acid receptor responder (tazarotene induced) 1 (RARRES1), transcript variant 2, mRNA [NM_002888]	RARRES1	4.93	hs|3q25.32	Hs.131269
Homo sapiens interferon, alpha-inducible protein 27 (IFI27), transcript variant 2, mRNA [NM_005532]	IFI27	4.92	hs|14q32.12	Hs.532634
Homo sapiens low density lipoprotein receptor class A domain containing 2 (LDLRAD2), mRNA [NM_001013693]	LDLRAD2	4.91	hs|1p36.12	Hs.710255
Homo sapiens tissue factor pathway inhibitor 2 (TFPI2), mRNA [NM_006528]	TFPI2	4.87	hs|7q21.3	Hs.438231
Homo sapiens LY6/PLAUR domain containing 5 (LYPD5), transcript variant B, mRNA [NM_182573]	LYPD5	4.85	hs|19q13.31	Hs.44289
Homo sapiens solute carrier organic anion transporter family, member 2B1 (SLCO2B1), transcript variant 1, mRNA [NM_007256]	SLCO2B1	4.83	hs|11q13.4	Hs.7884
Homo sapiens hypothetical LOC150568 (LOC150568), non-coding RNA [NR_015399]	LOC150568	4.83	hs|2q12.1	Hs.107284
Homo sapiens RAS, dexamethasone-induced 1 (RASD1), transcript variant 1, mRNA [NM_016084]	RASD1	4.80	hs|17p11.2	Hs.25829
Homo sapiens vascular cell adhesion molecule 1 (VCAM1), transcript variant 1, mRNA [NM_001078]	VCAM1	4.78	hs|1p21.2	Hs.109225
Homo sapiens triggering receptor expressed on myeloid cells 1 (TREM1), mRNA [NM_018643]	TREM1	4.76	hs|6p21.1	Hs.283022
Homo sapiens contactin 3 (plasmacytoma associated) (CNTN3), mRNA [NM_020872]	CNTN3	4.76	hs|3p12.3	Hs.12723
Homo sapiens zinc finger protein 323 (ZNF323), transcript variant 1, mRNA [NM_030899]	ZNF323	4.75	hs|6p22.1	Hs.656413
Homo sapiens centrosomal protein 192kDa pseudogene (LOC643201), non-coding RNA [NR_036494]	LOC643201	4.73	hs|5q35.2	Hs.390285
Homo sapiens chromosome 11 open reading frame 53 (C11orf53), mRNA [NM_198498]	C11orf53	4.73	hs|11q23.1	Hs.298685
Homo sapiens potassium voltage-gated channel, Isk-related family, member 4 (KCNE4), mRNA [NM_080671]	KCNE4	4.72	hs|2q36.1	Hs.348522
Homo sapiens nucleosome assembly protein 1-like 3 (NAP1L3), mRNA [NM_004538]	NAP1L3	4.72	hs|Xq21.32	Hs.21365
Homo sapiens solute carrier family 6 (neurotransmitter transporter, L-proline), member 7 (SLC6A7), mRNA [NM_014228]	SLC6A7	4.71	hs|5q32	Hs.241597
Homo sapiens LY6/PLAUR domain containing 1 (LYPD1), transcript variant 1, mRNA [NM_144586]	LYPD1	4.71	hs|2q21.2	Hs.432395
Homo sapiens chemokine (C-X-C motif) ligand 6 (granulocyte chemotactic protein 2) (CXCL6), mRNA [NM_002993]	CXCL6	4.67	hs|4q13.3	Hs.164021
Homo sapiens microtubule associated tumor suppressor 1 (MTUS1), transcript variant 2, mRNA [NM_001001925]	MTUS1	4.66	hs|8p22	Hs.7946
Homo sapiens platelet endothelial aggregation receptor 1 (PEAR1), mRNA [NM_001080471]	PEAR1	4.66	hs|1q23.1	Hs.142003
Homo sapiens aldo-keto reductase family 1, member C1 (dihydrodiol dehydrogenase 1; 20-alpha (3-alpha)-hydroxysteroid dehydrogenase) (AKR1C1), mRNA [NM_001353]	AKR1C1	4.65	hs|10p15.1	Hs.460260
Homo sapiens ankyrin repeat domain 57 pseudogene (LOC389834), non-coding RNA [NR_027420]	LOC389834	4.64	hs|21p11.2	Hs.720653
Homo sapiens NmrA-like family domain containing 1 pseudogene (LOC344887), non-coding RNA [NR_033752]	LOC344887	4.63	hs|3q27.2	Hs.128803
Homo sapiens cholecystokinin (CCK), transcript variant 1, mRNA [NM_000729]	CCK	4.62	hs|3p22.1	Hs.458426
Homo sapiens hypothetical LOC400550 (LOC400550), transcript variant 2, non-coding RNA [NR_036444]	LOC400550	4.60	hs|16q24.1	Hs.448825
Homo sapiens annexin A10 (ANXA10), mRNA [NM_007193]	ANXA10	4.60	hs|4q32.3	Hs.188401
Homo sapiens inositol hexakisphosphate kinase 3 (IP6K3), transcript variant 1, mRNA [NM_054111]	IP6K3	4.60	hs|6p21.31	Hs.17253
Homo sapiens parathyroid hormone 2 receptor (PTH2R), mRNA [NM_005048]	PTH2R	4.58	hs|2q34	Hs.570296
Homo sapiens cDNA FLJ11710 fis, clone HEMBA1005149. [AK021772]	FLJ11710	4.58	hs|17q22	Hs.657294
Homo sapiens T cell receptor gamma variable 7 pseudogene, mRNA (cDNA clone IMAGE:5210958). [BC027954]	TRGV7	4.57	hs|7p14.1	Hs.534032
Homo sapiens v-myb myeloblastosis viral oncogene homolog (avian) (MYB), transcript variant 2, mRNA [NM_005375]	MYB	4.56	hs|6q23.3	Hs.606320
Homo sapiens heparanase (HPSE), transcript variant 1, mRNA [NM_006665]	HPSE	4.56	hs|4q21.23	Hs.44227
Homo sapiens tudor domain containing 1 (TDRD1), mRNA [NM_198795]	TDRD1	4.55	hs|10q25.3	Hs.333132
Homo sapiens protein tyrosine phosphatase, receptor type, C (PTPRC), transcript variant 4, mRNA [NM_080923]	PTPRC	4.54	hs|1q31.3	Hs.654514
Homo sapiens serpin peptidase inhibitor, clade A (alpha-1 antiproteinase, antitrypsin), member 3 (SERPINA3), mRNA [NM_001085]	SERPINA3	4.53	hs|14q32.13	Hs.534293
Homo sapiens musashi homolog 2 (Drosophila) (MSI2), transcript variant 1, mRNA [NM_138962]	MSI2	4.48	hs|17q22	Hs.658922
Homo sapiens transforming growth factor, beta 2 (TGFB2), transcript variant 2, mRNA [NM_003238]	TGFB2	4.46	hs|1q41	Hs.133379
Homo sapiens potassium channel tetramerisation domain containing 4 (KCTD4), mRNA [NM_198404]	KCTD4	4.46	hs|13q14.12	Hs.23406
Homo sapiens apolipoprotein D (APOD), mRNA [NM_001647]	APOD	4.46	hs|3q29	Hs.522555
aldo-keto reductase family 1, member C1 (dihydrodiol dehydrogenase 1; 20-alpha (3-alpha)-hydroxysteroid dehydrogenase) [Source:HGNC Symbol;Acc:384] [ENST00000380859]		4.45	hs|10p15.1	
Homo sapiens leptin receptor (LEPR), transcript variant 3, mRNA [NM_001003679]	LEPR	4.44	hs|1p31.3	Hs.723178
Homo sapiens family with sequence similarity 155, member A (FAM155A), mRNA [NM_001080396]	FAM155A	4.43	hs|13q33.3	Hs.598102
