# Supplementary material for: Angiopoietin-Like 7 Is an Anti-Angiogenic Protein Required to Prevent Vascularization of the Cornea
Source: PLoS One. 2015 Jan 26;10(1):e0116838. doi: 10.1371/journal.pone.0116838 (PMC4306551; doi:10.1371/journal.pone.0116838)
Supplement: S2 Table — (PDF) [file pone.0116838.s002.PDF]

Relative downregulated genes in HCKs

Official Full Name	Gene Symbol	Fold change	Location	Unigene ID
Homo sapiens synaptophysin-like 2 (SYPL2), mRNA [NM_001040709]	SYPL2	-10.60	hs|12q13.13	Hs.658823
Homo sapiens aggrecan (ACAN), transcript variant 2, mRNA [NM_013227]	ACAN	-9.62	hs|8q12.3	Hs.591870
Homo sapiens alpha-2-macroglobulin (A2M), mRNA [NM_000014]	A2M	-9.44	hs|12q21.31	Hs.259559
Homo sapiens HOXA11 antisense RNA 1 (non-protein coding) (HOXA11-AS1), antisense RNA [NR_002795]	HOXA11-AS1	-9.30	hs|17q21.2	Hs.296942
Homo sapiens heat shock 27kDa protein 3 (HSPB3), mRNA [NM_006308]	HSPB3	-9.27	hs|3q22.3	Hs.289292
Homo sapiens homeobox C4 (HOXC4), transcript variant 1, mRNA [NM_014620]	HOXC4	-9.24	hs|17q21.2	Hs.296942
Homo sapiens homeobox C9 (HOXC9), mRNA [NM_006897]	HOXC9	-9.13	hs|14q21.1	Hs.248160
Homo sapiens fibrillin 2 (FBN2), mRNA [NM_001999]	FBN2	-9.11	hs|2q37.3	Hs.471783
Homo sapiens solute carrier family 8 (sodium/calcium exchanger), member 1 (SLC8A1), transcript variant A, mRNA [NM_021097]	SLC8A1	-9.04	hs|12p13.31	Hs.212838
Homo sapiens sema domain, immunoglobulin domain (Ig), short basic domain, secreted, (semaphorin) 3D (SEMA3D), mRNA [NM_152754]	SEMA3D	-8.98	hs|7p15.2	Hs.110637
Homo sapiens collagen, type XV, alpha 1 (COL15A1), mRNA [NM_001855]	COL15A1	-8.82	hs|10p12.1	Hs.128193
Homo sapiens indolethylamine N-methyltransferase (INMT), transcript variant 2, mRNA [NM_001199219]	INMT	-8.82	hs|6q27	Hs.487200
Homo sapiens homeobox A10 (HOXA10), transcript variant 1, mRNA [NM_018951]	HOXA10	-8.79	hs|7p22.3	Hs.159142
Homo sapiens early B-cell factor 3 (EBF3), mRNA [NM_001005463]	EBF3	-8.76	hs|5p13.2	Hs.591742
Homo sapiens insulin-like growth factor 2 (somatomedin A) (IGF2), transcript variant 1, mRNA [NM_000612]	IGF2	-8.71	hs|4q31.3	Hs.481022
Homo sapiens ATPase, Na+/K+ transporting, alpha 2 polypeptide (ATP1A2), mRNA [NM_000702]	ATP1A2	-8.57	hs|22q13.1	Hs.276808
Homo sapiens keratin 34 (KRT34), mRNA [NM_021013]	KRT34	-8.45	hs|9p24.1	Hs.446083
Homo sapiens forkhead box Q1 (FOXQ1), mRNA [NM_033260]	FOXQ1	-8.44	hs|4q21.21	Hs.369448
Homo sapiens stathmin-like 2 (STMN2), transcript variant 2, mRNA [NM_007029]	STMN2	-8.40	hs|12p13.31	Hs.212838
Homo sapiens secreted frizzled-related protein 2 (SFRP2), mRNA [NM_003013]	SFRP2	-8.39	hs|9q22.33	Hs.409034
Homo sapiens protein tyrosine phosphatase, receptor type, D (PTPRD), transcript variant 1, mRNA [NM_002839]	PTPRD	-8.37	hs|6p25.3	Hs.484423
Homo sapiens homeobox A9 (HOXA9), mRNA [NM_152739]	HOXA9	-8.34	hs|12q13.2	
Homo sapiens chordin-like 1 (CHRDL1), transcript variant 1, mRNA [NM_001143981]	CHRDL1	-8.27	hs|6p25.3	Hs.484423
Homo sapiens T-box 15 (TBX15), mRNA [NM_152380]	TBX15	-8.22	hs|12p13.31	Hs.212838
Homo sapiens C-type lectin domain family 3, member B (CLEC3B), mRNA [NM_003278]	CLEC3B	-8.17	hs|13q12.12	Hs.37167
Homo sapiens SPARC related modular calcium binding 2 (SMOC2), transcript variant 1, mRNA [NM_022138]	SMOC2	-7.99	hs|12p13.31	Hs.212838
Homo sapiens homeobox C6 (HOXC6), transcript variant 2, mRNA [NM_153693]	HOXC6	-7.97	hs|3q22.3	Hs.289292
Homo sapiens forkhead box L2 (FOXL2), mRNA [NM_023067]	FOXL2	-7.87	hs|2p23.1	Hs.567598
Homo sapiens somatostatin receptor 1 (SSTR1), mRNA [NM_001049]	SSTR1	-7.86	hs|2p22.1	Hs.468274
Homo sapiens homeobox A11 (HOXA11), mRNA [NM_005523]	HOXA11	-7.85	hs|11p15.5	Hs.272259
Homo sapiens chromosome 9 open reading frame 125 (C9orf125), mRNA [NM_032342]	C9orf125	-7.60	hs|12q24.21	Hs.381715
Homo sapiens LFNG O-fucosylpeptide 3-beta-N-acetylglucosaminyltransferase (LFNG), transcript variant 1, mRNA [NM_001040167]	LFNG	-7.52	hs|8q21.13	Hs.521651
Homo sapiens paired-like homeodomain 1 (PITX1), mRNA [NM_002653]	PITX1	-7.52	hs|10q26.3	Hs.591374
Homo sapiens Fraser syndrome 1 (FRAS1), transcript variant 1, mRNA [NM_025074]	FRAS1	-7.42	hs|17q21.2	Hs.296942
Homo sapiens limb bud and heart development homolog (mouse) (LBH), mRNA [NM_030915]	LBH	-7.41	hs|4q31.3	Hs.481022
Homo sapiens interleukin 7 receptor (IL7R), mRNA [NM_002185]	IL7R	-7.41	hs|1q23.2	Hs.34114
Homo sapiens chromosome 3 open reading frame 72 (C3orf72), mRNA [NM_001040061]	C3orf72	-7.37	hs|21q21.3	Hs.58324
Homo sapiens cyclin D2 (CCND2), mRNA [NM_001759]	CCND2	-7.35	hs|8q22.1	Hs.492277
Homo sapiens family with sequence similarity 162, member B (FAM162B), mRNA [NM_001085480]	FAM162B	-7.32	hs|3q22.3	Hs.289292
Homo sapiens olfactomedin-like 2A (OLFML2A), mRNA [NM_182487]	OLFML2A	-7.27	hs|12q13.13	Hs.549040
Homo sapiens paired box 3 (PAX3), transcript variant PAX3D, mRNA [NM_181458]	PAX3	-7.23	hs|9q22.33	Hs.409034
Homo sapiens homeobox A13 (HOXA13), mRNA [NM_000522]	HOXA13	-7.22	hs|9q31.1	Hs.655738
Homo sapiens collagen, type XXI, alpha 1 (COL21A1), mRNA [NM_030820]	COL21A1	-7.19	hs|12q21.1	Hs.170563
Homo sapiens tetraspanin 8 (TSPAN8), mRNA [NM_004616]	TSPAN8	-7.19	hs|5q11.2	Hs.41707
Homo sapiens G protein-coupled receptor 133 (GPR133), mRNA [NM_198827]	GPR133	-7.17	hs|5q31.1	Hs.84136
Homo sapiens Meis homeobox 1 (MEIS1), mRNA [NM_002398]	MEIS1	-7.14	hs|9q22.33	Hs.409034
Homo sapiens receptor (G protein-coupled) activity modifying protein 1 (RAMP1), mRNA [NM_005855]	RAMP1	-7.11	hs|9q22.33	Hs.409034
Homo sapiens matrix-remodelling associated 5 (MXRA5), mRNA [NM_015419]	MXRA5	-7.08	hs|9q22.33	Hs.409034
Homo sapiens mohawk homeobox (MKX), mRNA [NM_173576]	MKX	-7.00	hs|9q22.33	Hs.409034
Homo sapiens homeobox C8 (HOXC8), mRNA [NM_022658]	HOXC8	-6.98	hs|7p15.2	Hs.249171
Homo sapiens mannosyl (beta-1,4-)-glycoprotein beta-1,4-N-acetylglucosaminyltransferase (MGAT3), transcript variant 1, mRNA [NM_002409]	MGAT3	-6.95	hs|9q22.33	Hs.409034
Homo sapiens sarcoglycan, gamma (35kDa dystrophin-associated glycoprotein) (SGCG), mRNA [NM_000231]	SGCG	-6.94	hs|9q22.33	Hs.409034
Homo sapiens v-kit Hardy-Zuckerman 4 feline sarcoma viral oncogene homolog (KIT), transcript variant 2, mRNA [NM_001093772]	KIT	-6.91	hs|6p25.3	Hs.484423
Homo sapiens early B-cell factor 1 (EBF1), mRNA [NM_024007]	EBF1	-6.91	hs|12p13.32	Hs.376071
Homo sapiens ISL LIM homeobox 2 (ISL2), mRNA [NM_145805]	ISL2	-6.88	hs|6p25.3	Hs.484423
Homo sapiens wingless-type MMTV integration site family member 2 (WNT2), transcript variant 1, mRNA [NM_003391]	WNT2	-6.81	hs|15q26.1	Hs.2159
Homo sapiens growth differentiation factor 6 (GDF6), mRNA [NM_001001557]	GDF6	-6.80	hs|3q22.3	Hs.289292
Homo sapiens endothelin 1 (EDN1), transcript variant 1, mRNA [NM_001955]	EDN1	-6.77	hs|17q21.2	Hs.296942
Homo sapiens T-box 5 (TBX5), transcript variant 1, mRNA [NM_000192]	TBX5	-6.75	hs|12q13.13	Hs.549040
Homo sapiens ADAM metallopeptidase with thrombospondin type 1 motif, 5 (ADAMTS5), mRNA [NM_007038]	ADAMTS5	-6.72	hs|19p13.3	Hs.155597
Homo sapiens mab-21-like 1 (C. elegans) (MAB21L1), mRNA [NM_005584]	MAB21L1	-6.70	hs|6p25.3	Hs.591352
Homo sapiens microfibrillar associated protein 5 (MFAP5), mRNA [NM_003480]	MFAP5	-6.69	hs|6p25.3	Hs.484423
Homo sapiens ADAM metallopeptidase with thrombospondin type 1 motif, 8 (ADAMTS8), mRNA [NM_007037]	ADAMTS8	-6.68	hs|11q24.3	Hs.271605
Homo sapiens complement factor D (adipsin) (CFD), mRNA [NM_001928]	CFD	-6.66	hs|15q24.3	Hs.444677
Homo sapiens basic helix-loop-helix family, member e22 (BHLHE22), mRNA [NM_152414]	BHLHE22	-6.64	hs|6p24.1	Hs.511899
Homo sapiens acyl-CoA synthetase short-chain family member 3 (ACSS3), mRNA [NM_024560]	ACSS3	-6.62	hs|3q22.3	Hs.289292
Homo sapiens ubiquitin-like 4B (UBL4B), mRNA [NM_203412]	UBL4B	-6.59	hs|7p15.2	Hs.587427
Homo sapiens homeobox A7 (HOXA7), mRNA [NM_006896]	HOXA7	-6.56	hs|7p15.2	Hs.249171
Homo sapiens wingless-type MMTV integration site family, member 11 (WNT11), mRNA [NM_004626]	WNT11	-6.55	hs|12p13.31	Hs.212838
Homo sapiens adenylate cyclase 1 (brain) (ADCY1), mRNA [NM_021116]	ADCY1	-6.54	hs|12p13.31	Hs.512842
Homo sapiens heparan sulfate (glucosamine) 3-O-sulfotransferase 6 (HS3ST6), mRNA [NM_001009606]	HS3ST6	-6.50	hs|16p13.3	Hs.670144
Homo sapiens R-spondin 3 homolog (Xenopus laevis) (RSPO3), mRNA [NM_032784]	RSPO3	-6.49	hs|6q22.33	Hs.135254
Homo sapiens heat shock 70kD protein 12B (HSPA12B), transcript variant 1, mRNA [NM_052970]	HSPA12B	-6.44	hs|20p13	Hs.516854
Homo sapiens protein phosphatase 1, regulatory (inhibitor) subunit 14A (PPP1R14A), mRNA [NM_033256]	PPP1R14A	-6.39	hs|19q13.2	Hs.631569
Homo sapiens chromosome 8 open reading frame 85 (C8orf85), mRNA [NM_001025357]	C8orf85	-6.39	hs|8q24.11	Hs.437551
Homo sapiens hox transcript antisense RNA (non-protein coding) (HOTAIR), antisense RNA [NR_003716]	HOTAIR	-6.33	hs|12q13.13	Hs.197076
Homo sapiens chemokine (C-X-C motif) ligand 14 (CXCL14), mRNA [NM_004887]	CXCL14	-6.32	hs|5q31.1	Hs.483444
Homo sapiens heart and neural crest derivatives expressed 2 (HAND2), mRNA [NM_021973]	HAND2	-6.31	hs|4q34.1	Hs.388245
Homo sapiens phosphoglucomutase 5 (PGM5), mRNA [NM_021965]	PGM5	-6.30	hs|9q21.11	Hs.307835
Homo sapiens lipid phosphate phosphatase-related protein type 4 (LPPR4), transcript variant 1, mRNA [NM_014839]	LPPR4	-6.26	hs|1p21.2	Hs.13245
Homo sapiens protein phosphatase 1, regulatory (inhibitor) subunit 9A (PPP1R9A), transcript variant 4, mRNA [NM_017650]	PPP1R9A	-6.22	hs|7q21.3	Hs.21816
Homo sapiens short stature homeobox 2 (SHOX2), transcript variant 1, mRNA [NM_003030]	SHOX2	-6.21	hs|3q25.32	Hs.55967
Homo sapiens doublecortin-like kinase 1 (DCLK1), transcript variant 1, mRNA [NM_004734]	DCLK1	-6.20	hs|13q13.3	Hs.507755
Homo sapiens cartilage intermediate layer protein, nucleotide pyrophosphohydrolase (CILP), mRNA [NM_003613]	CILP	-6.19	hs|15q22.31	Hs.442180
Homo sapiens microtubule-associated protein 6 (MAP6), transcript variant 1, mRNA [NM_033063]	MAP6	-6.14	hs|11q13.5	Hs.585540
Homo sapiens homeobox C10 (HOXC10), mRNA [NM_017409]	HOXC10	-6.10	hs|12q13.13	Hs.44276
Homo sapiens MDS1 and EVI1 complex locus (MECOM), transcript variant 2, mRNA [NM_005241]	MECOM	-6.09	hs|3q26.2	Hs.656395
Homo sapiens family with sequence similarity 19 (chemokine (C-C motif)-like), member A5 (FAM19A5), transcript variant 2, mRNA [NM_015381]	FAM19A5	-6.08	hs|22q13.32	Hs.436854
Homo sapiens tet oncogene 1 (TET1), mRNA [NM_030625]	TET1	-6.03	hs|10q21.3	Hs.567594
Homo sapiens cytokine receptor-like factor 1 (CRLF1), mRNA [NM_004750]	CRLF1	-6.00	hs|19p13.11	Hs.114948
Homo sapiens family with sequence similarity 38, member B (FAM38B), mRNA [NM_022068]	FAM38B	-6.00	hs|18p11.22	Hs.436902
Homo sapiens armadillo repeat containing 4 (ARMC4), mRNA [NM_018076]	ARMC4	-5.99	hs|10p12.1	Hs.127530
Homo sapiens transmembrane protein 30B (TMEM30B), mRNA [NM_001017970]	TMEM30B	-5.99	hs|14q23.1	Hs.146180
Homo sapiens odd-skipped related 2 (Drosophila) (OSR2), transcript variant 2, mRNA [NM_053001]	OSR2	-5.98	hs|8q22.2	Hs.253247
Homo sapiens chromosome 7 open reading frame 69 (C7orf69), mRNA [NM_025031]	C7orf69	-5.97	hs|7p12.3	Hs.287647
Homo sapiens transmembrane protein 176B (TMEM176B), transcript variant 1, mRNA [NM_014020]	TMEM176B	-5.97	hs|7q36.1	Hs.647090
Homo sapiens LIM domain binding 2 (LDB2), transcript variant 1, mRNA [NM_001290]	LDB2	-5.94	hs|4p15.32	Hs.714330
Homo sapiens adrenergic, alpha-2A-, receptor (ADRA2A), mRNA [NM_000681]	ADRA2A	-5.91	hs|10q25.2	Hs.249159
Homo sapiens RAB38, member RAS oncogene family (RAB38), mRNA [NM_022337]	RAB38	-5.88	hs|11q14.2	Hs.591975
Homo sapiens TLR4 interactor with leucine-rich repeats (TRIL), mRNA [NM_014817]	TRIL	-5.87	hs|7p14.3	Hs.21572
